# Opportunities and Challenges of Nanoparticles in Digestive Tumours as Anti-Angiogenic Therapies

**DOI:** 10.3389/fonc.2021.789330

**Published:** 2022-01-10

**Authors:** Zhengyang Yang, Wei Deng, Xiao Zhang, Yongbo An, Yishan Liu, Hongwei Yao, Zhongtao Zhang

**Affiliations:** Department of General Surgery, Beijing Friendship Hospital, Capital Medical University and National Clinical Research Center for Digestive Diseases, Beijing, China

**Keywords:** digestive tumours, angiogenesis, anti-angiogenesis, nanoparticles, therapy

## Abstract

Digestive tumours, a common kind of malignancy worldwide, have recently led to the most tumour-related deaths. Angiogenesis, the process of forming novel blood vessels from pre-existing vessels, is involved in various physiological and pathological processes in the body. Many studies suggest that abnormal angiogenesis plays an important role in the growth, progression, and metastasis of digestive tumours. Therefore, anti-angiogenic therapy is considered a promising target for improving therapeutic efficacy. Traditional strategies such as bevacizumab and regorafenib can target and block the activity of proangiogenic factors to treat digestive tumours. However, due to resistance and some limitations, such as poor pharmacokinetics, their efficacy is not always satisfactory. In recent years, nanotechnology-based anti-angiogenic therapies have emerged as a new way to treat digestive tumours. Compared with commonly used drugs, nanoparticles show great potential in tumour targeted delivery, controlled drug release, prolonged cycle time, and increased drug bioavailability. Therefore, anti-angiogenic nanoparticles may be an effective complementary therapy to treat digestive tumours. In this review, we outline the different mechanisms of angiogenesis, the effects of nanoparticles on angiogenesis, and their biomedical applications in various kinds of digestive tumours. In addition, the opportunities and challenges are briefly discussed.

## Introduction

The human digestive system consists of digestive gland organs (salivary glands, liver, and pancreas) and digestive tubes (oral cavity, pharynx, oesophagus, stomach, small intestine, large intestine, and rectum). Digestive tumours, principally hepatocellular carcinoma, pancreatic cancer, oesophageal cancer, gastric cancer, and colorectal cancer, lead to the greatest number of tumour-related deaths worldwide (1, 2). Moreover, digestive tumours accounted for 43.3% of the cancer incidence from 2000 to 2015 in China (3). The current therapeutic strategies for digestive tumours mainly consist of surgical resection, chemotherapy, radiotherapy, molecular targeting therapy, and immunotherapy. Because of indefinite clinical symptoms, deficient imaging features, and sensitive biomarkers, most patients are diagnosed at an advanced stage with an unsatisfactory 5-year survival rate (4, 5). Chemotherapy, including neoadjuvant and postoperative therapy, which is currently the primary approach to treat such patients, cannot achieve gratifying curative effects because of the multidrug resistance mechanisms in tumours (6, 7). Therefore, novel therapeutic strategies are required to better treat patients with digestive tumours.

Angiogenesis is the formation of novel blood vessels from pre-existing vessels and is a highly regulated process (8–10). Judah Folkman, considered the father of angiogenesis research, advanced the notion in 1971 that tumour growth depends on angiogenesis, which is essential for removing metabolites, supplying oxygen and nutrients, and promoting the metastatic ability of cancer cells (11, 12). Additionally, Folkman proposed that the tumour size would be limited to less than 2 mm^3^ in the absence of angiogenesis and would then enter a dormant state, thus raising the possibility of using anti-angiogenic antibodies for the treatment of cancers (13, 14). Although a variety of anti-angiogenic drugs, such as bevacizumab and sunitinib, were approved worldwide in the following half-century and have certain effectiveness, adaptive resistance and some adverse events associated with poor pharmacokinetics have limited the further application of this therapy (15, 16). The mechanisms for anti-angiogenic therapeutic resistance have been widely reported mainly including direct effects of hypoxia (co-option of normal vessels in paracancerous tissues, vascular mimicry, and induction of tumour invasion and metastasis), the influence of tumour stromal cells (recruitment of tumour-associated macrophages, endothelial progenitor cells, and pro-angiogenic myeloid cells), and upregulating alternative pro-angiogenic factors (17–19). Additionally, some tumour cells have been reported that could continuously grow without angiogenesis, which might result in the resistance of anti-angiogenic therapies (20, 21). Thus, exploring novel anti-angiogenic tactics to surmount the resistance and side effects to achieve better therapeutic effects is urgent.

The rapid advancement of nanotechnology has brought about more opportunities for anti-angiogenic therapies to treat digestive tumours. Due to the highly leaky blood vasculature and absence of functional lymphatic vessels in solid tumours, nanoparticles (20–200 nm in diameter) could avoid immune clearance, further prolonging their half-life and specifically accumulating in tumour tissues, called the enhanced permeability and retention (EPR) effect (22–24). Thus, nanotechnology-based medicine, also called nanomedicine, has made many advances in cancer treatment, especially in the areas of targeted delivery of drugs and medical imaging (25, 26). Moreover, nanoparticles could also solve the aforementioned shortcomings of current conventional anti-angiogenic therapies. In fact, nanoparticles have demonstrated great advantages as anti-angiogenic drugs through targeted delivery, controlled release, prolonged half-life, and increased bioavailability. However, due to their dissimilar physicochemical properties, different nanoparticles possess corresponding features of biodistribution properties and half-lives (27, 28). Therefore, this article aimed to summarize the different mechanisms of angiogenesis and the actual applications of anti-angiogenic nanoparticles in digestive tumours and discuss the current opportunities and challenges.

## Mechanisms of Angiogenesis

Angiogenesis primarily consists of four sequential steps: I) dissolution of extracellular matrix components surrounding blood vessels like basement membrane glycoproteins by proteolytic enzymes; II) activation and migration of endothelial cell; III) proliferation of endothelial cell; and IV) formation of capillary tubes (29). However, when the balance between anti-angiogenic factors and pro-angiogenic factors is broken in some pathological conditions (like asthma, atherosclerosis, myocardial ischaemia, hypertension, and tumour progression), angiogenic activators will be upregulated, further resulting in aberrant angiogenesis (30).

### Mechanisms of Tumour Angiogenesis

In 1971, Folkman proposed that tumours could not grow more than 2 mm^3^ without vascular supply because of insufficient oxygen and nutrition supply and poor clearance of metabolic waste, which would further cause hypoxia or acidosis (31). The physiological angiogenic process is maintained under the dynamically relative homeostasis, which is being referred to as “angiogenic switch” (32). Once this homeostasis is disrupted in tumours, the “angiogenic switch” will be active, and the vascular endothelial cells will be affected to upregulate the secretion of angiogenic promoters and downregulate the secretion of angiogenic inhibitors (33, 34). Over the past 50 years, the complex mechanisms of tumour angiogenesis have been exposed with more intensified researches.

Different types of angiogenic regulators could be released from tumour cells, blood, endothelial cells, and extracellular matrix (35, 36). Currently reported angiogenic promoters include vascular endothelial growth factor (VEGF), epidermal growth factor (EGF), transforming growth factor (TGF), fibroblast growth factor (FGF), angiopoietin-1 and -2 (ANG-1 and -2), and platelet-derived growth factor (PDGF), while angiogenic inhibitors include angiostatin; endostatin; platelet factor-4; tissue inhibitors of metalloproteinases (TIMPs); thrombospondin-1 (TSP-1); interferon (IFN)-α, -β, and -γ; and interleukin (IL)-12 (37). Some biological pathways like metabolic stress (hypoxia, hypoglycaemia, and lower pH), gene mutation (activation of oncogenes and inactivation of anti-oncogene), inflammatory response (tissue inflammatory infiltration), and mechanical stress (interactions by proliferating cells) can turn on the “angiogenic switch”, further resulting in tumourigenesis (38, 39). Among these pathways, hypoxia plays an important role in driving tumour angiogenesis, which can stimulate the expression of angiogenic stimulating factors in cancer cells. The transcriptional programs mediated by hypoxia-inducible factor (HIF) can activate hypoxia, which acts as a central regulator of detection and adaptive oxygen levels. HIF promotes the overexpression of VEGF A (VEGFA) and its receptors VEGF receptor-1 and -2 (VEGFR-1 and -2) (16). Moreover, HIF can promote the expression of ANG-2, which helps the proliferation of endothelial cells in hypoxic tumour areas, further destroying the integrity of the vascular wall (40). Tumour hypoxic conditions can also upregulate the expression of PDGF, which conducts as the mitogen of fibroblast and mesenchymal cells and induces different angiogenic actions (41). Additionally, matrix metalloproteinases (MMPs) can degrade the extracellular matrix, further mediating various changes in tumour microenvironment to advance the angiogenic process (42).

### Conventional Anti-Angiogenic Therapy and Limitations

**Figure 1:**
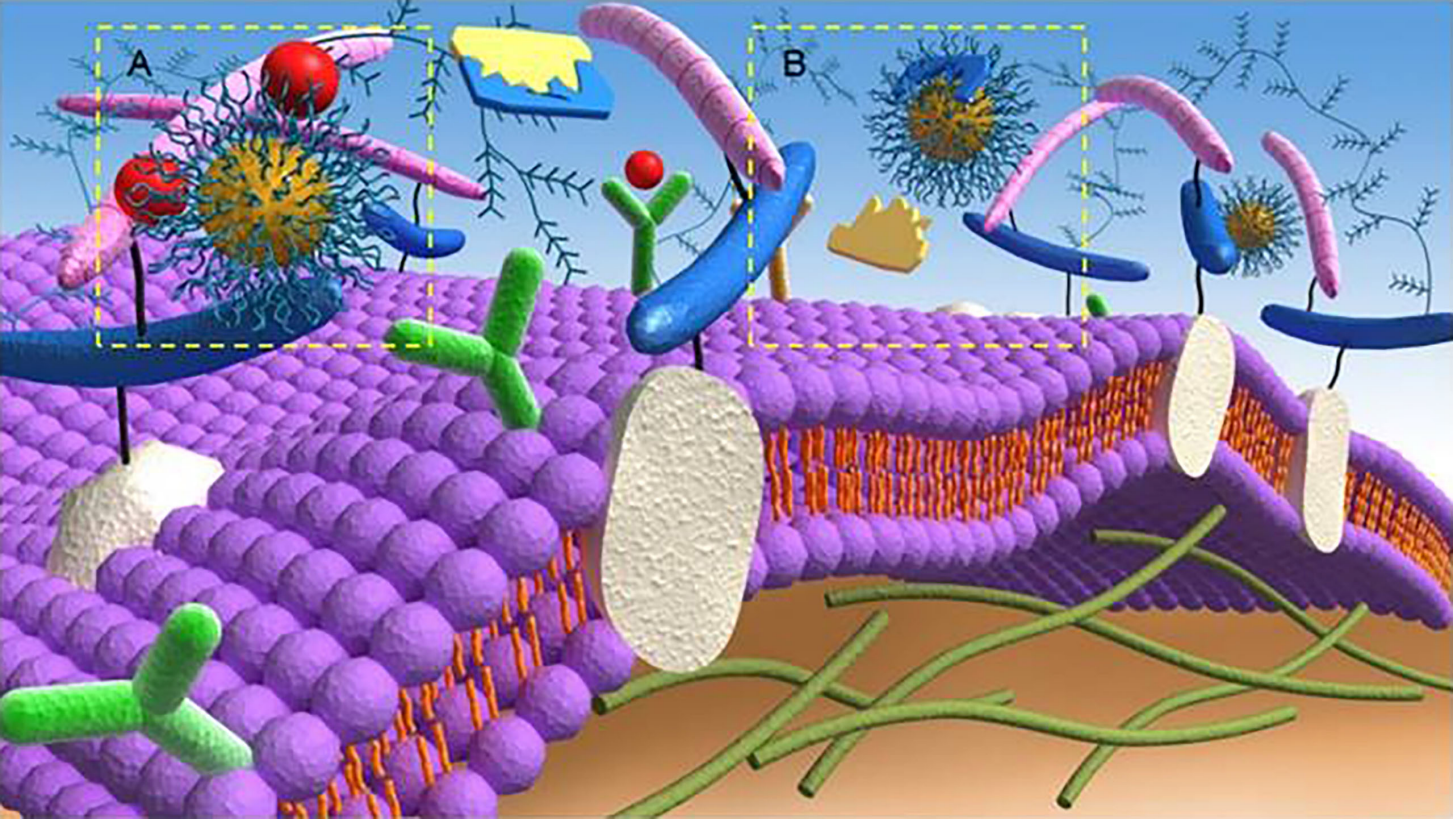
Anti-angiogenic mechanisms of targeting **(A)** angiogenic growth factors and **(B)** proteolytic enzymes of the extracellular matrix by anti-angiogenic strategies. Reproduced with permission (42). Copyright 2018, Ivyspring International Publisher.

In the past 20 years, various anti-angiogenic agents were developed and prolonged the survival time of patients to some extent. Among them, more than 10 anti-angiogenic agents have been approved for the treatment of different digestive malignancies (**Table S1** of the Supplementary Material) (43, 44). The mechanism of such agents is to prevent tumour cells from obtaining nutrition by restricting available blood vessels and blocking the formation of novel blood vessels in tumour sites. Briefly, the mechanisms of most existing anti-angiogenic strategies include blocking the interactions of VEGF and VEGFR to their respective receptors (**Figure 1**) (43). It was also reported that metronomic chemotherapy, which was defined as using small doses of the high-frequency chemotherapeutic drug to achieve a lower but effective range of drug concentrations over long periods without significant toxicity, could downregulate VEGF, further upregulating the expression of TSP-1 to play an important role in inhibiting tumour angiogenic dormancy (45–47)[a-c]. However, these therapies often have adverse reactions like drug resistance, toxicity, and even thrombotic and haemorrhagic diseases (48).

Despite that anti-angiogenic therapies sometimes stabilize diseases and prolong survival, such treatment might lead to more drug-resistant tumours and a higher patient recurrence rate. Such clinical harm might be related to the compensatory upregulation of angiogenic factors, further promoting tumour angiogenesis and tumour escape mechanism, which lead to acquired drug resistance. Among them, hypoxia plays an important role in tumour resistance to anti-angiogenic therapies and leads to more aggressive metastatic diseases with worse prognosis. Hypoxia-related HIF-1 pathway plays an important role in the resistance to anti-angiogenic therapies and is the main survival factor for cancer cells to overcome the hypoxic environment. Hypoxia is reported to regulate hepatocyte growth factor/mesenchymal–epithelial transition factor (HGF/c-MET) signal pathway, further activating mitogen-activated protein kinases/extracellular signal-regulated kinase (MAPK/ERK) cascades, phosphoinositide 3-kinase/protein kinase B/mammalian target of rapamycin (PI3K/Akt/mTOR) pathway, and so on to promote tumourigenesis, progression, and drug resistance (49–52)[y3-y6]. In the phase III METEOR trial, advanced renal cell carcinoma (RCC) patients after previous VEGFR-targeted therapy were given cabozantinib (tyrosine kinase inhibitor), which could raise the survival rate (53)[y7].

For example, the anti-angiogenic efficacy of bevacizumab might be greatly weakened by the alternative pro-angiogenic signals generated during tumour proliferation and metastasis (46). The hypoxic microenvironment produced in the anti-angiogenic process could induce HIF-1 α and stimulate the expression of β1 integrin, which had been upregulated in bevacizumab-resistant tumours. Meanwhile, targeting β integrin could enhance anti-angiogenic therapies and inhibit the growth of bevacizumab-resistant tumours in xenograft models (54)[y8]. Additionally, many preclinical and phase I/II clinical trials have shown that a single anti-angiogenic strategy cannot effectively inhibit tumour growth, which promoted the development of multi-drug combination therapies (55). However, in addition to causing serious side effects, combination therapies usually performed poor biodistribution and pharmacokinetic characteristics (56).

With the development of science and technology, the field of nanobiology has attracted increasing attention in recent years. Nanotechnology-based medicine, also known as nanomedicine, has promoted tremendous advances in cancer treatment, especially in the areas of targeted drug delivery and medical imaging. Meanwhile, nanomedicine has made remarkable achievements in the research and development of drug development for clinical tumour treatment. Among them, many nanobased drugs have been used in the clinical chemotherapy of multiple gastrointestinal tumours, such as colorectal cancer, including doxorubicin liposomes, paclitaxel liposomes, and albumin-bound paclitaxel (27). The drugs mentioned above not only can improve the local treatment concentration but also can significantly reduce the non-specific toxicity of organs. Moreover, they can solve the problem of allergies to solvents (such as castor oil) caused by the poor water solubility of some common chemotherapy drugs (such as paclitaxel), further improving the quality of life of patients (57). These nanoparticles take different easily modified and highly biocompatible materials as their main body (such as organic compounds, proteins, lipids, and polymers), which are rationally modified and designed to be multifunctional drugs to achieve specific imaging and precise treatment of tumours (58).

## Mechanism and Superiority of Nanoparticles

**Figure 2:**
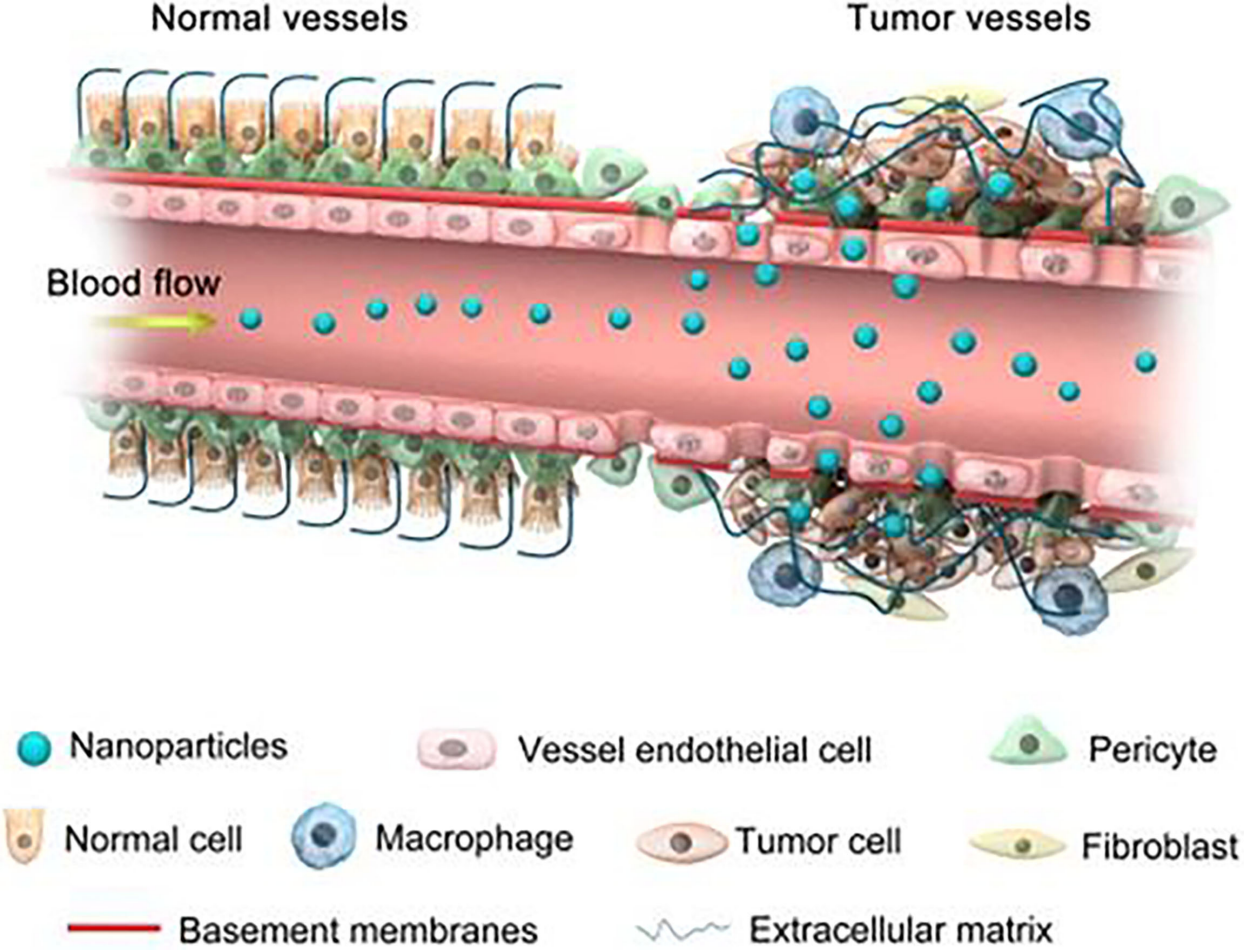
Schematic diagram of nanoparticles targeting tumour tissues through enhanced permeability and retention (EPR) effect. Normal blood vessels are composed of dense endothelial cells, which can prevent the escape and extravasation of nanoparticles. Blood vessels of tumour tissue are leaky and highly permeable, allowing the preferential accumulation of nanoparticles in the interstitial space of the tumour. Reproduced with permission (42). Copyright 2018, Ivyspring International Publisher.

Generally, nanomedicine is defined as a technology used to study the properties and potential applications of materials sized 20–200 nm (59). Compared with traditional drugs, nanoparticles have unique properties and advantages, such as a large surface area, to achieve a high drug loading rate, easy surface modification to add new functions, protection of drugs from degradation or metabolism, controlled release of drugs, and passive accumulation in tumours (60). They have the potential to modulate the pharmacokinetic and pharmacodynamic characteristics of drugs to increase their therapeutic concentration. Compared with traditional drug delivery methods, intravenous administration of nanoparticles can passively accumulate drugs in tumour tissues through the EPR effect, further improving the drug concentration in the tumour site, which is realized based on the special histopathological characteristics of tumour tissue (61). Briefly, normal blood vessels are composed of dense endothelial cells, which can prevent the escape and extravasation of nanoparticles. However, the blood vessels of tumours are leaky and highly permeable, resulting in the preferential accumulation of nanoparticles in tumour tissues (**Figure 2**) (43). In addition, targeting molecules can be modified on the surface of nanoparticles to bind to highly expressed receptors on the surface of tumour cells to play an active targeting role of tumour tissue (62). Nanoparticles are increasingly widely considered for the diagnosis and treatment of tumours because of their important properties of passive and active targeting.

### Passive Targeting of Nanoparticles

Passive-targeting nanomaterials, which can be transported into tumour tissues through the EPR effect, are also the most widely studied nanoparticle drug delivery systems at present. To ensure that the tumour tissues receive sufficient nutrients and oxygen to facilitate rapid tumour growth, the blood vessels of most solid tumours have structural defects and produce a large number of vascular permeability factors so that most tumours exhibit high vascular permeability (63). The phenomenon produced by this special pathological anatomy that can promote the accumulation of nanomaterial substances in tumour tissues is called the EPR effect, which mainly manifests as macromolecules with molecular weights greater than 40 kDa selectively leaking out of tumour tissues and accumulating in solid tumours but not in normal tissues (64). The EPR effect is mainly related to the size of nanoparticles. Briefly, when the diameter is less than 4 nm, nanoparticles are not only likely to be filtered through the glomerulus in the systemic circulation and then discharged through the kidney but also diffuse back into the blood circulation after entering the tumour tissue because of the large pores between the vascular endothelium, which reduces the passive-targeting effect.

Nanoparticles greater than 400 nm in diameter are easily swallowed up by the reticuloendothelial system as foreign matter during blood circulation. Even if they reach the blood vessels near the tumour, they cannot permeate into tumour tissues because they are larger than the blood vessels. That is why the EPR effect is best when the size of the nanoparticles is from 20 to 200 nm (65–67). The EPR effect based on the characteristics of solid tumours is a milestone for tumour-targeted drug delivery and has been widely used in the development of nanobased antitumour drugs.

### Stimulus-Responsive Nanoparticles

Stimulus-responsive nanoparticles achieve targeted delivery and controlled release of drugs by responding to the stimulation of physics, chemistry, and biomolecules (68). Their advantage is that they can realize the controlled release of drugs through the stimulation of trigger conditions, further reduce the loss of drugs during the process of blood circulation and reduce the toxicity of drugs (69).

It is well known that tumour tissue has a lower pH than normal tissue because cells in normal tissue are powered by oxidative phosphorylation, while in tumour tissue, cells are powered by glycolysis, resulting in the production of large amounts of lactic acid, which is known as the Warburg effect (70). The pH value in normal tissues is close to neutral (7.4), while in most tumour cells, it is slightly acidic (≤6.5) (70). Nanoparticles are prepared using pH-dependent chemical bonds to make them stable under physiological conditions. In a weakly acidic tumour environment, the chemical bonds can break, release the drugs, and improve the local accumulation rate of drugs in the tumour site (71).

According to the literature, cancer stromal cells actively secrete glutathione (GSH), resulting in a concentration of GSH in tumour cells (2~10 mmol/L) 100~1,000 times that in normal cells (2~20 μmol/L) and 100 times that in normal tissue, resulting in a strongly reducing environment in colorectal cancer (72, 73). Due to the existence of the mercaptan group in GSH, it can act as a reducing agent and become an important antioxidant, further decomposing some essential chemical bonds such as disulfide bonds and diselenide bonds (74, 75). Therefore, GSH stimulation-responsive nanoparticles are widely used for targeted delivery and controlled release of antitumour drugs. In addition, temperature, magnetic force, light, electric field, force, ATP, DNA, RNA, and enzymes can also be used as factors to stimulate drug release in nanoparticles, further improving the tumour treatment efficiency (76, 77).

### Active Targeting of Nanoparticles

Active-targeting nanomaterials, a novel approach to antitumour nanotechnology, can specifically bind to receptors overexpressed on the surface of tumours and tumour vascular endothelial cells by modifying the corresponding ligands on the surface of nanoparticles. Active targeting mainly depends on the interaction between ligand molecules and the surface receptors of tumour cells. Therefore, the ideal ligands used for active targeted delivery of antitumour drugs should be able to bind to tumour cells as much as possible but not to normal cells. A variety of ligands have been used for active antitumour drug targeted delivery, including folic acid, glucose, peptides, proteins, antibodies, and small interfering RNAs (siRNAs) (78, 79). The advantage of active targeting nanocarriers is that off-target effects can be avoided as much as possible (80). In summary, the aim of active targeting nanomedicine is to achieve a high affinity between receptors and ligands. Compared with passive targeting, the active targeted nanomaterial delivery system can enhance the binding of nanomaterials to tumour cells, reduce the non-specific uptake of nanomaterials, avoid the generation of drug resistance, and increase the distribution of drugs at the tumour site (81). In addition, nanocarriers have a variety of drug delivery capabilities, such as timely administration of chemotherapeutic drugs, targeted drugs, prodrugs, and drug kinases (82).

### Imaging Diagnosis

Because different tumour stages require corresponding treatment methods, the treatment of digestive tumours largely depends on accurate imaging diagnostic technology. CT, MRI, and PET are the most commonly used imaging techniques for diagnosing digestive tumours in the clinic. Nevertheless, these techniques are mainly based on the histomorphology and metabolic changes of tumours, which exhibit poor sensitivity in some cases, such as micrometastasis and small tumours (83). Nanoparticles can wrap high concentrations of imaging agents such as iodine, magnetic materials, and radioactive substances inside themselves to amplify the signals generated by tumours. In addition, nanocarriers can weaken the signal intensity of normal tissue, further reducing any interference with the diagnosis (84, 85).

In addition, new technologies such as near-infrared (NIR) fluorescence imaging can be used to observe the tissue morphology and metabolism, providing additional possibilities to detect important anatomical structures and tumour lymph node metastasis in real-time during surgery (86). The wavelength of NIR fluorescence is from 700 to 900 nm, with high tissue penetration (at the centimetre level) (87). After packaging NIR agents into nanoparticles and injection, the fluorescence signal can be captured by a laparoscopic fluorescent imaging system in real time, while human eyes are not sensitive to the NIR wavelength, and their presence will not change the surgical field.

### Synergistic Therapy

Phototherapy, a light-mediated therapy, has gradually attracted increasing attention recently because of its advantages of minimal invasiveness, spatiotemporal controllability, and low toxicity in tumour treatment. Phototherapy includes photodynamic therapy (PDT) and photothermal therapy (PTT). PDT mainly relies on photosensitizers to absorb energy under light conditions, causing a series of photochemical and photobiological reactions and producing cytotoxic substances such as reactive oxygen species (ROS), which selectively damage tumour tissues (88). Indocyanine green (ICG), which has been approved by the Food and Drug Administration (FDA) for intraoperative fluorescence imaging in the clinic, is currently widely studied as a PDT agent due to its antitumour effects (89). The principle of PTT is that the photothermal medium converts light energy into heat energy after being irradiated by a laser, causing an increase in the local tissue temperature to achieve the killing effect (90). PTT has a wide antitumour spectrum because the process does not need oxygen. Additionally, most PTT agents are excited by NIR lasers, which can penetrate deeply into tissues and kill more tumour cells (91).

A synergistic system of phototherapy and chemotherapy was constructed through nanotechnology, in which the improvement of vascular permeability caused by phototherapy could increase the accumulation of nanoparticles in tumours, further enhancing the effect of chemotherapy (92). The thermal effect induced by PTT not only promotes the release of drugs by the nanoparticles but also changes the permeability of the cell membrane, further increasing the endocytosis of cancer cells to chemotherapeutic drugs (93). Nanotechnology can integrate different therapeutic functions into single nanoparticles, achieving a more thorough treatment mode and bringing new ideas and hope for tumour treatment. Moreover, nanoparticles can achieve tumour theranostics, which means that effective treatment is performed at the same time as tumour diagnosis, while the curative effect is monitored by diagnostic methods at the same time (94).

## Anti-Angiogenic Nanoparticles in Digestive Tumours

**Figure 3:**
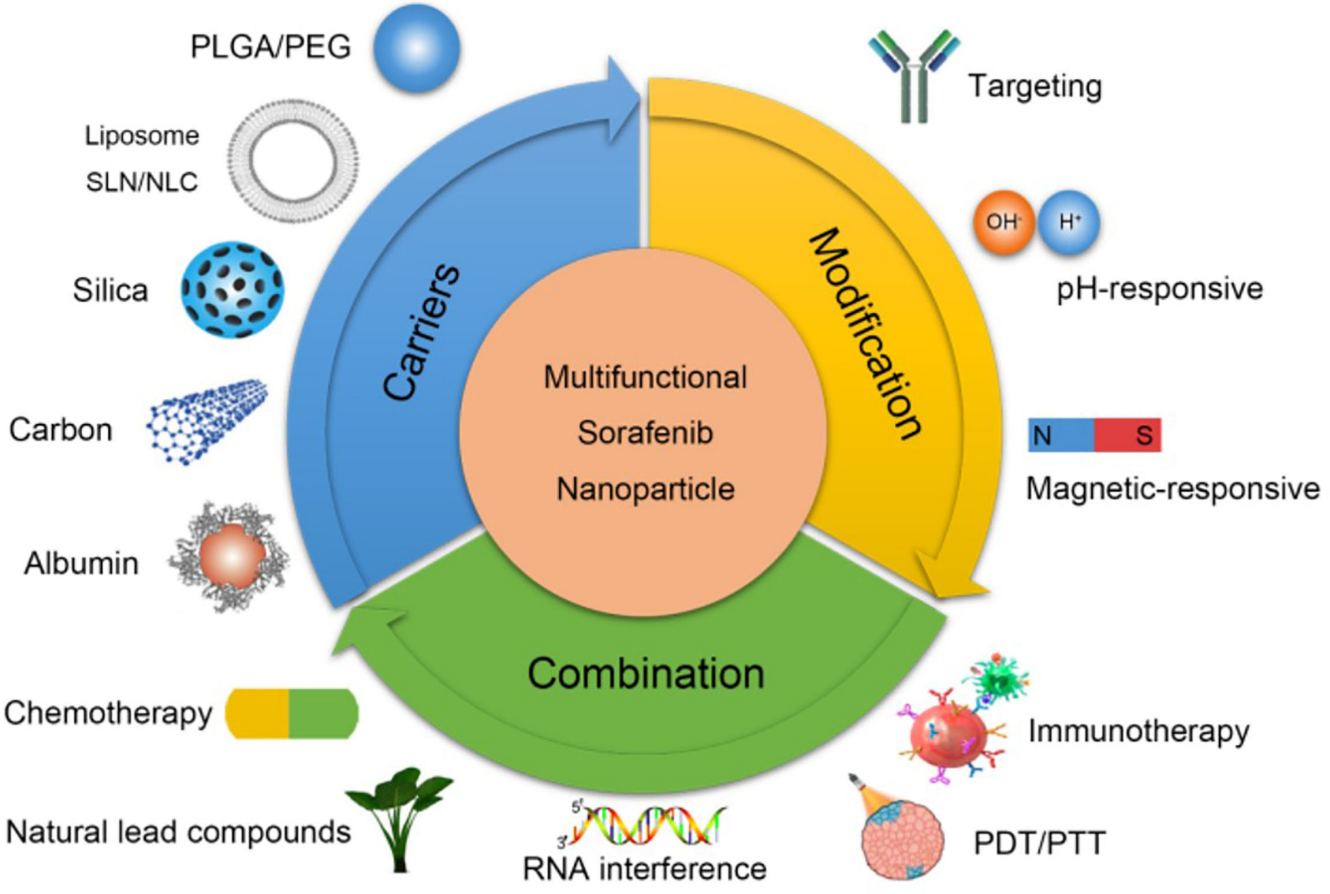
Mechanisms of anti-angiogenic nanoparticles in hepatocellular carcinoma. Improving the biocompatibility of anti-angiogenic agents through different nanocarriers (top left). Increasing the targeting and responsiveness of anti-angiogenic agents through modification methods (top right). Enhancing curative effect and overcoming resistance through combined with other therapies (bottom) (95). Copyright 2018, Ivyspring International Publisher.

Based on the advantages mentioned above, researchers had developed many novel nanoparticles to overcome the drug resistance of anti-angiogenic therapies in digestive tumours. The fundamental mechanisms of enhanced anti-angiogenic treatment through nanoparticles are shown in **Figure 3** (95). Nanoparticles loaded with anti-angiogenic drugs had high drug release efficiency and bioavailability, which could actively target tumour tissues. As for some drugs with poor solubility, nanocarriers could provide better delivery characteristics through liposome coating. In addition, nanoparticles can specifically control drug release through surface modification, effectively reduce the therapeutic dose and administration frequency, and further reduce the cytotoxicity and adverse reactions of chemotherapeutic drugs. For example, some nanoparticles could be transferred to tumour tissues *in vivo* through a magnetic field and then respond to acidic tumour environment for releasing loaded drugs (96). More importantly, combining anti-angiogenic therapies with other targeted therapeutic drugs and/or immunotherapy could effectively reduce resistances by blocking their occurrence mechanism. Co-delivering anti-angiogenic agents and hypoxia-specific siRNA through nanoparticles, the most critical step of tumour resistance (hypoxia) was inhibited to defeat drug resistance and acquire a better therapeutic effect (97, 98). Additionally, tumour-targeted nanoparticles co-delivered by oxygen-generating MnO_2_ and sorafenib could decompose H_2_O_2_ to oxygen to alleviate hypoxia-driven drug resistance further enhance anti-angiogenic effect and provide benefits to digestive tumours treatment (99). The reported anti-angiogenic nanotherapeutics are described below, which we are looking forward to overcoming the limitations of the current strategies, further improving their antitumour therapeutic outcomes in digestive tumours.

### Metal and Metallic Compounds in Nanoparticles

Metal nanoparticles have been widely considered with various applications in digestive tumours for anti-angiogenic treatment. Among them, gold (Au) nanoparticles are considered one of the most appropriate therapeutic options against tumours, and they have the advantages of chemical stability, small dimensions, low cytotoxicity, and inherent biocompatibility (100). Additionally, some studies have indicated that Au nanoparticles (AuNPs) possess anti-angiogenic properties. Mukherjee et al. first reported in 2005 that Au nanoparticles could specifically bind to VEGF-165 and basic FGF, further resulting in inhibition of endothelial and fibroblast cell proliferation *in vitro* as well as VEGF-induced permeability and angiogenesis *in vivo*. In addition, such nanoparticles exhibited no significant hepatic or renal toxicity in tumour-bearing mice (101). Based on the superior PTT characteristics of Au nanoparticles, CD44v6-GNSs (Au nanoparticle-conjugated CD44v6 monoclonal antibodies) were constructed. Such CD44v6-GNSs could inhibit the growth of gastric cancer and remarkably extend survival in tumour-bearing mice. Moreover, photoacoustic imaging indicated that CD44v6-GNSs could specifically target the gastric cancer vascular system after intravenous injection *in vivo* (102).

Furthermore, Au nanoparticles possess considerable advantages as carriers for targeted drug delivery. 5-Fluorouracil (5-FU), a thymidylate synthase inhibitor, is a commonly used chemotherapeutic drug against colorectal cancer with various side effects, such as bone marrow suppression, anorexia, and vomiting (103). Liszbinski et al. loaded 5-FU on Au nanoparticles coated with anti-EGFR (EGF receptor) antibodies to treat colorectal cancer. Such AuNP-5FU-EGFR nanoparticles showed superior efficiency in apoptosis induction over single 5-FU with no significant cytotoxic effects in human colorectal cancer cells (104). Delivering siRNA to tumour tissues has always been a great challenge. Because of their higher molecular weight and polyanionic properties, naked siRNAs would be degraded swiftly by serum ribonucleases, causing difficulties in crossing cellular membranes (105). It was reported that Au nanoparticles might be an appropriate and safe choice to deliver siRNA. A novel sequence of siRNA that targeted the oncogene c-Myc was designed and bound to branched polyethylenimine (bPEI)-modified Au nanoparticles. Such siRNA/bPEI/AuNPs could effectively deliver siRNA into human hepatoma cells and successfully silence the c-Myc gene with no significant cytotoxicity (106). Interestingly, the expression of the c-Myc gene was positively correlated with the expression of proangiogenic-related genes in many digestive tumours, including hepatocellular carcinoma, pancreatic cancer, and colorectal cancer (107–109).

Radiation therapy is a common clinical treatment for digestive tumours and could be used as a supplement to neoadjuvant therapy or as an auxiliary mean to prevent postoperative tumour recurrence. As a high atomic number element, Au could cause tumour tissue to have a mass energy coefficient and a higher atomic number than normal tissue while targeting the tumour region, further improving the treatment rate of radiotherapy. Au nanoparticles could enhance the efficacy of radiotherapy by regulating the cell cycle, inducing DNA damage, producing oxidative stress, and potentially interfering with bystander effects (110). Alhussan et al. functionalized Au nanoparticles with polyethylene glycol (PEG) and arginine-glycine-aspartate (RGD), the ligand for integrins, to acquire the GNP_PEG-RGD_ complex. GNP_PEG-RGD_ could not only target pancreatic cancer cells but also be used as a drug carrier and radiosensitizing agent. Moreover, the uptake of GNP_PEG-RGD_ by cancer-associated fibroblasts (CAFs) could be 10% higher than that of pancreatic cancer cells, causing the targeted killing of CAFs and achieving an antitumour effect (111). As a significant stromal cell component, CAFs could promote angiogenesis in digestive tumours by upregulating proangiogenic factors and controlling the biomechanical properties of the tumour matrix, such as elasticity, stiffness, and interstitial fluid pressure (112, 113). There are also studies reporting that biodegradable honeycomb-like gold nanoparticles (HGNs) could act as both radiosensitizing agents and photothermal agents to achieve synergistic photothermal radiotherapy. Such an approach could help nanoparticles accumulate more efficiently to improve the oxygen supply and damage double-stranded DNA in the tumour tissues of xenograft pancreatic cancer mice (114).

As a trace element, copper (Cu) plays an important role in multiple biological processes, such as oxidative metabolism, angiogenesis, tumourigenesis, metastasis, and relapse, while its imbalance can cause various diseases (115). A retrospective study demonstrated that a higher serum copper level was associated with relapse or disease progression in haematological malignancies. In addition, it was positively related to some adverse prognostic markers in chronic lymphocytic leukaemia, such as an increased percentage of unmutated IgVH and higher expression of ZAP70 and CD38 (116). Bai et al. reported hollow copper sulfide (CuS) nanoparticles encapsulating sorafenib and surface modified with anti-VEGFR antibodies. While CuS-SF@CMV nanoparticles kill hepatoma cells by CuS-mediated PTT, sorafenib and anti-VEGFR antibodies inhibit tumour angiogenesis through the PI3K/AKT and Ras/Raf/MEK/ERK pathways to achieve continuous inhibition against tumour metastasis (117). Cui et al. modified the surface of CuS with PEG and cyclic RGDfK peptide [c(RGDfK)] to acquire CuS-PEG-c(RGDfK) nanoparticles, which not only possessed the property of selective tumour uptake but also significantly killed hepatoma cells through thermal ablation (118). There are also studies reporting that cetuximab was modified on CuS to acquire active targeting of CuS nanoparticles (CuS-Ab NPs) with excellent PTT efficacy and superior biocompatibility in xenograft models (119).

Silver (Ag) nanoparticles are another important therapeutic noble metal widely used in medical applications. Gurunathan et al. first reported that Ag nanoparticles could have anti-angiogenic potential by inhibiting the VEGF-induced PI3K/Akt cell survival signal in bovine retinal endothelial cells (120). It was also reported that Ag nanoparticles could inhibit the process of angiogenesis by exhibiting dose-dependent cytotoxic effects on endothelial cells (121). Ag nanoparticles also had effective antitumour activities in lung cancer, melanoma, cervical cancer, breast cancer, and lymphoma cell lines (122–124). Given the lack of relevant research, additional animal experiments and preclinical studies are expected to validate the effectiveness of Ag nanoparticles in treating digestive tumours.

Superparamagnetic iron oxide nanoparticles (SPIONs) are widely used as targeted drug carriers with superior biocompatibility, good chemical stability, and low toxicity (125). Wang et al. loaded gambogic acid onto magnetic Fe_3_O_4_ nanoparticles (MNP-Fe_3_O_4_) called GA-MNP-Fe_3_O_4_, which inhibited the migration and proliferation of pancreatic cancer cells and downregulated the downstream target gene of angiogenesis, VEGF (126). It has also been reported that using hyaluronate (HA)- and trimethyl chitosan (TMC)-recoated SPIONs could significantly prevent the angiogenesis of colorectal cancer cells (127). Additionally, SPIONs modified with vasculature-specific binding peptides could potentially be used to observe the angiogenic status of gastric cancer *in vivo* (128).

### Non-Metallic Nanoparticles

With the advantages of a large relative surface area, adjustable pore size, higher drug loading efficiency, easy functionalization, and good biocompatibility, silica-based nanoparticles have been widely used as drug delivery systems in nanomedicine (129). In addition, silicate nanoparticles have been found to have potential anti-angiogenic effects against retinal neovascularization. Jo et al. demonstrated that intravitreal injection of silicate nanoparticles could effectively reduce anomalous retinal angiogenesis in retinopathy mice without direct toxicity. The specific mechanism might be that such nanoparticles could inhibit VEGF-related angiogenesis by suppressing VEGFR-2 phosphorylation and blocking the activation of ERK (130). Additionally, Setyawati et al. confirmed that silica nanoparticles could inhibit the proliferation, migration, invasion, and viability of endothelial cells, further restraining angiogenesis by triggering the production of intracellular ROS and activating the p53 gene-related pathway. This study also reported that compared with 40- and 100-nm nanoparticles, nanoparticles with a diameter of 60 nm exhibited the most effective inhibitory effect against angiogenesis (131). In another study, mesoporous silica nanoparticles (MSNs) were used to encapsulate evodiamine (EVO) and berberine (BBR) to acquire a delivery platform with temperature and pH responsiveness. This dual drug delivery platform exhibited excellent synergistic therapeutic effects against angiogenesis, cell migration, and invasion in hepatoma and colon cancer cells (132). Fluorescent silica nanoparticles marked by endoglin aptamers have been demonstrated to interfere with the TGF-β pathway by binding to tumour vascular endothelial cell membrane proteins, further inhibiting angiogenesis and reducing vascular density in xenograft hepatocellular carcinoma mice (133). Silica-based nanoparticles have also been reported to have anti-angiogenic potential in pancreatic and colorectal cancers (134–136).

Carbon is the second most abundant element in the body, and the application of carbon-based nanoparticles such as graphene, nanodiamonds, carbon nanotubes, and carbon nanodots in the antitumour, especially anti-angiogenic, field has been widely studied recently (137). Murugesan et al. reported first that carbon-based nanoparticles such as graphite, multiwalled carbon nanotubes, and fullerenes exhibited remarkable anti-angiogenic activity against both FGF- and VEGF-induced angiogeneses in a chick chorioallantoic membrane model (138). Lai et al. found that bovine serum albumin-capped graphene oxide (BSA-GO) could strongly bind to VEGF-A_165_ and act as an effective angiogenesis inhibitor. Such nanoparticles could thereby block the interaction of VEGF-A_165_ with the VEGF receptor and stop the downstream signalling pathway of angiogenesis in hepatoma cells (139).

Recently, Ding et al. developed PEI-modified single-walled carbon nanotubes (SWNTs) to deliver VEGF-targeted siRNA (siVEGF) for synergistic targeted treatment against angiogenesis. The observations in xenograft pancreatic adenocarcinoma mice indicated that such nanoparticles could significantly accumulate in tumour tissues and inhibit the growth and angiogenesis of the tumours. Moreover, low cytotoxicity, good biocompatibility, and negligible organ toxicity were observed in this study (140). However, the renal clearance, toxicology, and biocompatibility of carbon-based nanoparticles are still controversial and limit their further application. Some studies have suggested that they might penetrate the cell membranes of healthy tissue, resulting in harmful inflammatory and fibrotic responses and cell death (141, 142).

### Polymeric Nanoparticles and Liposomes

Synthetic and naturally derived polymeric nanoparticles have also received great attention in various biomedical fields, especially in drug delivery systems for cancer treatment and other diseases (143). As a naturally alkaline polysaccharide, chitosan has been widely used as a candidate material for drug carriers, taking advantage of its biodegradability, lower immunogenicity, better biocompatibility, and non-toxicity (144). Chitosan-based nanoparticles have been widely used in the treatment of digestive tumours, including anti-angiogenic therapies. Zhang et al. designed *N*-deoxycholic acid-glycol chitosan (DGC) as a carrier loaded with the commonly used chemotherapeutic agent docetaxel (DCT) and the angiogenic marker peptide for gastric cancer (GX1) to obtain multifunctional vascular targeting nanoparticles (GX1-DGC-DCT). GX1 could effectively promote the uptake of nanoparticles by cells, as observed by confocal laser scanning microscopy. After intravenous injection of GX1-DGC-DCT, tumour growth in xenograft gastric cancer models showed a tumour inhibition rate of 67.05% compared with the single DCT group (145). Some similar studies in gastric cancer reported that chitosan oligosaccharide (COS)-conjugated selenium (Se) and carboxymethyl chitosan (CMCS)-conjugated norcantharidin (NCTD) could remarkably enhance its antitumour efficacy through regulating the VEGF-related pathway to repress angiogenesis with non-toxic effects (146, 147). In addition, the delivery of siRNA by chitosan-based nanoparticles was also studied extensively. Nikkhoo et al. reported carboxymethyl dextran-conjugated TMC (TMC-CMD) nanoparticles loaded with signal transducer and activator of transcription 3 (STAT3)-specific siRNA and BV6, a well-known inhibitor of apoptosis (IAP) inhibitor. The results showed that such nanoparticles could reduce both *in vitro* and *in vivo* tumour growth and angiogenesis by decreasing the expression of related genes, including TGF, VEGF, and FGF, in colorectal cancer (148). Some similar studies reported that chitosan-based nanoparticles loaded with IL-6-specific siRNA and HIF-1α-specific siRNA could inhibit colorectal cancer progression and angiogenesis (149, 150). Epirubicin (EPI), as an anthracycline derivative, is a first-line chemotherapy drug against various digestive tumours (151). Nasr et al. loaded EPI in chitosan nanoparticles to treat hepatocellular carcinoma, and it exhibited lower cardiotoxicity and superior results in reducing angiogenesis, overcoming resistance, and enhancing the therapeutic efficacy (152). Chitosan-based nanoparticles have also been reported as potential candidates for anti-angiogenic treatment in other digestive tumours, such as cholangiocarcinoma and pancreatic cancer (153–156).

PEG, polylactic acid (PLA), and polycaprolactone (PCL) are FDA-approved commercially available biodegradable copolymers that are widely used to prepare nanoparticles for drug delivery (157). Apatinib (a selective VEGFR-2 inhibitor) and DCT (Taxotere) are widely used for combined treatment in digestive tumours, but their curative effects appear to be impaired due to the disadvantages of their poor pharmacometabolic characteristics (158). Yu et al. constructed PEG-PCL liposomes as a drug delivery system for apatinib and DCT. They could achieve locally higher drug concentrations and prolong the release time, further decreasing angiogenesis, promoting apoptosis, and inhibiting proliferation in xenograft colorectal cancer mice (159). Liu et al. prepared PEG-PLA micelles to coencapsulate paclitaxel (PTX) and itraconazole (ITA) to produce PTX-ITA micelles (PIM) nanoparticles. PIM showed excellent systemic pharmacokinetics and increased drug accumulation in the tumour site. Additionally, PIM normalized blood vessels and inhibited tumour growth in both a human orthotopic pancreatic cancer model and genetically engineered spontaneous pancreatic ductal adenocarcinoma mice (160). Liposomal nanodelivery systems have also been widely studied in various digestive tumours, such as hepatocellular carcinoma and gastric cancer (161–163).

## Future Perspectives and Summary

Digestive tumours account for nearly half of the cancer incidence in China and cause the most tumour-related deaths worldwide. As a common molecular targeted therapy in the clinic, angiogenesis plays an important role in the development of digestive tumours. Anti-angiogenic therapies have been identified as an effective direction in digestive tumour treatment, but they are associated with some limitations, such as potential resistance and adverse reactions. Nanomaterials are considered superior tools to solve the above problems and achieve individual therapies. However, the successful translation of nanoparticles from the laboratory to the clinic could improve cancer treatment, but many challenges remain. In recent years, nanoparticles have been shown to possess stronger biocompatibility and more efficient tumour targeting capability through reasonable functionalization and modification. By adjusting their surface properties and the size and shape of nanoparticles, their drug toxicity and pharmacokinetics can be changed both *in vitro* and *in vivo* (164). In this article, we reviewed the biomedical applications of different nanoparticles, including metal/metallic compounds, non-metallic nanoparticles, polymeric nanoparticles, and liposomes, in various digestive tumours (**Table 1**). These nanoparticles can be used in various ways, such as delivering siRNA, antihypoxia, molecular targeting peptide phototherapy, and photothermal anti-angiogenic therapy.

**Table 1:** Representative nanoparticles mentioned in review for anti-angiogenic therapies in different digestive tumours.

Tumour Category	Design of Nanoparticles	Anti-Angiogenic Mechanism	Antitumour Outcome	Reference
Gastric cancer	Au nanoparticles conjugated CD44v6 monoclonal antibodies (CD44v6-GNS)	Specifically target gastric cancer neovascularization system for achieving photothermal therapy	Inhibit the growth of gastric cancer cells and extend the survivability remarkably of mice	(102)
Colorectal cancer	5-FU loaded on Au nanoparticles which were coated with anti-EGFR antibodies (AuNP-5FU-EGFR)	Specifically target EGFR positive tumour cells for enhancing the delivery of 5-FU antineoplastic agents	Superior efficiency on apoptosis induction than single 5-FU with no significant cytotoxic effects in colorectal cancer cells	(104)
Hepatocellular carcinoma	Polyethylenimine-modified Au nanoparticles were bound to siRNA, which targeted oncogene c-Myc (siRNA/bPEI/AuNPs)	Successfully silence c-Myc gene, which positively correlated with the expression of pro-angiogenic-related genes with no significant cytotoxicity	Enhance the cellular uptake of siRNA without significant cytotoxicity	(106)
Pancreatic cancer	Functionalize Au nanoparticles with polyethylene glycol and arginine-glycine-aspartate (GNP_PEG-RGD_)	Inhibit the cancer-associated fibroblasts related to angiogenesis	Increase the nanoparticles uptake by cancer-associated fibroblasts to kill such cells	(111)
Pancreatic cancer	Honeycomb-like gold nanoparticles mediated interventional photothermal therapy combined with brachytherapy (HGN-mediated IPT-BT)	Improve oxygen supply to overcome hypoxia-related resistance to anti-angiogenic therapies	Improve oxygen supply and damage double-stranded DNA in tumour tissues of xenograft pancreatic cancer mice	(114)
Hepatocellular carcinoma	Hollow copper sulfide nanoparticles encapsulating sorafenib and surface modified with anti-VEGFR antibodies (CuS-SF@CMV)	Inhibit tumour angiogenesis through PI3K/AKT and Ras/Raf/MEK/ERK pathways	Enhance synergistic PTT and chemotherapy against hepatoma cells through homotypic cell targeting and immune escape	(117)
Hepatocellular carcinoma	Modify the surface of CuS with PEG and cyclic RGDfK peptide (CuS-PEG-c(RGDfK))	Promote selective angiogenic tumour cells uptake of nanoparticles and kill such cells	Target nanoparticles to tumour vasculature and αvβ3 integrin-expressing tumour cells mediated efficient photothermal ablation of tumours	(118)
Pancreatic cancer	Load gambogic acid onto magnetic Fe_3_O_4_ nanoparticles (GA-MNP- Fe_3_O_4_)	Downregulate the downstream target gene of angiogenesis	Inhibit the migration and proliferation of cancer cells	(126)
Colorectal cancer	Recoat superparamagnetic iron oxide nanoparticles using hyaluronate and trimethyl chitosan (SPION-TMC-HA)	Block the initiator (HIF-1α) and end (EP4) of HIF-1α/COX2/PGE2/EP4 signalling pathways	Prevent proliferation, migration, invasion, angiogenesis, and colony formation of the cancer cells	(127)
Gastric cancer	Couple GEBP11 peptide to meso-2,3-dimercaptosuccinic acid-coated Fe_3_O_4_ magnetic nanoparticles and Cy5.5 fluorescent dye (Cy5.5-GEBP11-DMSA-MNPs, CGD-MNPs)	Target to tumour angiogenesis by coating novel vasculature-specific binding peptide, GEBP11	Could observe the angiogenic status of gastric cancer in xenograft cancer mice	(128)
Hepatocellular carcinoma and colorectal cancer	Encapsulate evodiamine and berberine through mesoporous silica nanoparticles	Response to tumour microenvironment and release the drugs for improving local drug concentration and biocompatibility	Exhibit excellent synergistic therapeutic effect against angiogenesis, cell migration and invasion in hepatoma and colon cancer cells	(132)
Hepatocellular carcinoma	Mark mouse endoglin aptamer, YQ26 to fluorescent silica nanoparticles (YQ26-FSiNPs)	Interfere with TGF-β pathway by binding to tumour vascular endothelial cell membrane protein, further inhibiting angiogenesis to reduce vascular density	Achieve prominently high targeting efficiency and therapeutic effects both *in vitro* experiments and *in vivo* animal studies	(133)
Pancreatic cancer	Polyethylenimine modified single-walled carbon nanotubes linked with candesartan to deliver VEGF targeted siRNA (SWNT−PEI−CD/siVEGF)	Deliver VEGF-targeted siRNA (siVEGF) for the synergistic and targeted treatment of tumour angiogenesis	Nanoparticles accumulate in tumour tissues and inhibit the growth and angiogenesis of tumour with low cytotoxicity and negligible organ toxicity	(140)
Gastric cancer	Load docetaxel and gastric cancer angiogenic marker peptide, GX1 through *N*-deoxycholic acid-glycol chitosan (GX1-DGC-DCT)	Decorated with GX1, which exhibited high affinity and specificity with the gastric cancer vasculature for targeted delivery hydrophobic docetaxel	Promote the uptake of nanoparticles in cells and inhibit tumour growth in xenograft gastric cancer models	(145)
Gastric cancer	Chitosan oligosaccharide conjugated selenium (COS–Se)	Reduce the expressions of CD34 and VEGF in treated tumour tissues	Inhibit proliferation and metastasis both *in vitro* and *in vivo*	(146)
Gastric cancer	Carboxymethyl chitosan conjugate norcantharidin (CNC)	Downregulate expressions of VEGF	Enhance the antitumour efficacy *in vivo*	(147)
Colorectal cancer	Carboxymethyl dextran-conjugated trimethyl chitosan (TMC-CMD)	Decrease angiogenesis-related genes expression including TGF, VEGF, and FGF	Reduce both *in vitro* and *in vivo* tumour growth and angiogenesis	(148)
Colorectal cancer	Polyethylene glycol chitosan lactate conjugated with hyaluronate and co-delivered anti-IL-6 siRNA (H-PCL-siRNA IL-6)	Co-delivery IAPs inhibitor (BV6) and anti-IL-6 siRNA by nanoparticles to achieve simultaneous therapy	Decrease cell migration, proliferation, colony formation, and angiogenesis in cancer cells and suppress cancer progression in xenograft colorectal cancer mice	(149)
Colorectal cancer	Carboxylated graphene oxide conjugated with trimethyl chitosan and hyaluronate to load HIF-1α-siRNA (siRNA loaded CGO-TMC-HA)	Suppress the CDKs/HIF-1α pathway-related resistance to anti-angiogenic therapies	Decrease the proliferation, migration, angiogenesis and colony formation in cancer cells	(150)
Hepatocellular carcinoma	Load epirubicin in chitosan nanoparticles (EPI-NPs)	Actively target tumour cells and release the drugs for superior efficacy and higher safety	Reduce angiogenesis, overcome resistance and enhance therapeutic efficacy with lower cardiotoxicity	(152)
Colorectal cancer	Polyethylene glycol-polycaprolactone liposome to deliver apatinib and docetaxel (Lipo-Apa and DOC)	Construct drug delivery system for the delivery of apatinib and docetaxel for synergistic therapy	Decrease angiogenesis, promote apoptosis and inhibit proliferation in xenograft colorectal cancer mice	(159)
Pancreatic cancer	Polyethylene glycol- polylactic acid micelle to coencapsulate paclitaxel and itraconazole (PIM)	Demonstrate optimized systemic pharmacokinetics and increase tumour drug accumulation due to serum stability	Increase drug accumulation, normalize blood vessels and inhibit tumour growth in a human orthotopic pancreatic cancer model	(160)

5-FU, 5-fluorouracil; EGFR, endothelial growth factor receptor; AuNP, Au nanoparticle; bPEI, branched polyethylenimine; HGN, honeycomb-like gold nanoparticle; IPT-BT, interventional photothermal–brachytherapy; VEGFR, vascular endothelial growth factor receptor; PTT, photothermal therapy; PEG, polyethylene glycol; SWNT, single-walled carbon nanotube.

Although nanoparticles have potential therapeutic effects, their application still has some limitations. The complexity and diversity of tumours and the different properties of nanoparticles would lead to different uptake *in vivo*. A better understanding of their intracellular transport and cellular uptake mechanisms might effectively reveal the potential therapeutic benefits of nanoparticles. Moreover, careful evaluation of their toxicity is required before the clinical applications of nanoparticles. Although various studies have proven that nanoparticles exhibit better biocompatibility and scarce cytotoxicity in preclinical models, their potential toxicity and an uncertain fate in the human body are still worrying. Therefore, developing more appropriate models to further evaluate the toxicity of nanoparticles is of great importance. In addition, nanoparticles also need to solve the phenomenon of drug resistance against anti-angiogenic therapies. Although some scientific and technical considerations are required before translational clinical applications, we have adequate reason to believe that combined with existing treatment strategies, anti-angiogenic therapies could become part of the treatment approach to digestive tumours.

## Author Contributions

All authors made substantial contributions to this review. ZZ and HY conceived and designed the review. ZY, WD, XZ, YA, and YL retrieved and reviewed the literatures. ZY, WD, and XZ wrote the manuscript. ZZ and HY reviewed and edited the manuscript. All authors read and approved the manuscript.

## Funding

This work was supported by grants from the National Key Technologies R&D Program (No. 2015BAI13B09), National Key Technologies R&D Program of China (No. 2017YFC0110904), and Clinical Center for Colorectal Cancer, Capital Medical University (No. 1192070313).

## Conflict of Interest

The authors declare that the research was conducted in the absence of any commercial or financial relationships that could be construed as a potential conflict of interest.

## Publisher’s Note

All claims expressed in this article are solely those of the authors and do not necessarily represent those of their affiliated organizations, or those of the publisher, the editors and the reviewers. Any product that may be evaluated in this article, or claim that may be made by its manufacturer, is not guaranteed or endorsed by the publisher.
