# Supplementary material for: Opportunities and Challenges of Nanoparticles in Digestive Tumours as Anti-Angiogenic Therapies
Source: Front Oncol. 2022 Jan 10;11:789330. doi: 10.3389/fonc.2021.789330 (PMC8784389; doi:10.3389/fonc.2021.789330)
Supplement: Supplementary file 1 [file DataSheet_1.docx]

**Supplementary Material**

**Supplementary table S1** Summary of anti-angiogenic agent for digestive cancer therapy.

| Anti-angiogenic agent | Indications in digestive tumours | Mechanism of action |
| --- | --- | --- |
| Bevacizumab | Metastatic colorectal cancer, hepatocellular carcinoma | Bind VEGF and prevent the interaction of VEGF to its receptors on the surface of endothelial cells |
| Sunitinib | Gastrointestinal stromal tumour, advanced pancreatic neuroendocrine tumours | Small molecule that inhibits multiple receptor tyrosine kinases, some of which are implicated in pathologic angiogenesis of cancer |
| Sorafenib | Hepatocellular carcinoma | Small molecule tyrosine kinase inhibitor while several of these kinases are thought to be involved in tumour angiogenesis |
| Cediranib | Recurrent colorectal cancer | Pan-VEGF tyrosine kinase inhibitor that inhibits all three VEGF receptors |
| Vatalanib | Metastatic colorectal cancer | Inhibit all three VEGF receptors tyrosine kinase signaling by competitively blocking the adenosine triphosphate-binding site |
| Brivanib | Hepatocellular carcinoma, colorectal cancer | Tyrosine kinase inhibitor which can inhibit both FGF and VEGF signaling |
| Ziv-aflibercept | Metastatic colorectal cancer | Bind to VEGF-A, VEGF-B and placental growth factor and inhibit the binding and activation of their cognate receptors |
| Regorafenib | Colorectal cancer, gastrointestinal stromal tumours and hepatocellular carcinoma | Small molecule inhibitor of multiple membrane-bound and intracellular kinases involved in pathologic tumour angiogenesis |
| Everolimus | Progressive neuroendocrine tumours of pancreatic origin | Inhibitor of mammalian target of rapamycin which can reduce the expression of VEGF |
| Surufatinib | Advanced pancreatic neuroendocrine tumour | Small-molecule inhibitor that targets tumour angiogenesis (VEGFR-1, VEGFR-2, VEGFR-3, and fibroblast growth factor receptor 1) |
| Lenvatinib | Unrespectable hepatocellular carcinoma | Kinase inhibitor that inhibits the kinase activities of VEGF receptors VEGFR-1, VEGFR-2 and VEGFR-3 |
| Pemigatinib | Previously treated, unrespectable locally advanced or metastatic cholangiocarcinoma | Small molecule kinase inhibitor that targets fibroblast growth factor receptor-1, -2 and -3 |
| Infigratinib | Previously treated, unrespectable locally advanced or metastatic cholangiocarcinoma | Small molecule kinase inhibitor of fibroblast growth factor receptor |
